# The Wear Performance of Cu-Based Composites Reinforced with Boron Nitride Nanosheets

**DOI:** 10.3390/ma15155282

**Published:** 2022-07-30

**Authors:** Changshun Zhu, Ruitao Li

**Affiliations:** School of Mechanical Engineering, Jiangsu University, Zhenjiang 212013, China; zcs@ujs.edu.cn

**Keywords:** copper matrix composites, boron nitride nanosheets, spark plasma sintering, wear performance

## Abstract

Copper matrix composites (CMCs) were prepared by blending Cu particles with boron nitride nanosheets (BNNSs) and then by consolidating the blended particles using spark plasma sintering (SPS). The relative density of the compacts was over 99%, and an intact interface was formed between Cu and the BNNSs. Within the range of the BNNS content studied, its introduction improved microhardness and wear resistance. With the introduction of 0.2 vol% BNNSs, the friction coefficient reduced from 0.15 to merely 0.07, and the wear resistance improved by over 100%. This makes the CMCs reinforced with BNNSs promising materials in applications such as bearings.

## 1. Introduction

Copper matrix composites (CMCs) have broad applications in aerospace, electronics and automotive fields due to their excellent electrical, thermal and tribological properties [1,2,3,4]. CMCs reinforced with graphite have widespread applications, such as brushes, friction parts and bearing materials due to their superb self-lubricating properties [2,5]. However, the porous structure of graphite makes it difficult to guarantee the mechanical properties of composites [6]. To reduce friction and wear, a large amount of graphite needs to be added to the copper matrix, which deteriorates the performance of CMCs. Therefore, a search for the suitable reinforcements of wear-resistant CMCs is of paramount importance.

Carbon nanomaterials, such as carbon nanotubes [7], graphene [3] and their derivatives [8], have been extensively studied as reinforcement phases in Cu matrix composites, owing to their inherent unique properties. The two-dimensional structure makes graphene more compatible with copper matrices, enhancing mechanical interlocking and load transfer with the matrices. Thus, graphene has been extensively studied as reinforcement for CMSs. For example, Chen et al. [9] reported that the mechanical performance of copper could be strengthened evidently by graphene addition mainly through load transfer and dislocation strengthening effects. Zhang et al. [10] fabricated a three-dimensional graphene-like network of reinforced CMC. Such an interpenetrating structural feature not only improves the interfacial shear stress but also promotes the crack-bridging toughening of the composite simultaneously.

Boron nitride nanosheets (BNNSs) have a similar structure to graphene with alternating boron and nitrogen atoms in the sp^2^ honeycomb network [11,12,13]. Such structural similarity makes some of their properties very close, such as mechanical strength and thermal conductivity. They are both excellent self-lubricating materials. However, N can form a stronger bond with Cu atoms than C in the interfacial region when BNNSs are used as reinforcements in a Cu matrix [14,15]. Specifically, BNNSs can have a stronger interfacial strength than graphene does. Additionally, BNNSs are more oxidation resistant than graphene [16]. This may endow BNNSs/Cu with some unique tribological properties, such as a high working temperature [17,18,19]. To the best of our knowledge, the tribological properties of BNNSs/Cu has seldom been investigated. Thus, it is of great interest to study the wear properties of CMCs reinforced with BNNSs.

To explore the full reinforcing potential of BNNSs, their content in CMCs should be well-determined, as demonstrated by other researchers. Liu et al. [20] has reported that 0.5 vol% BNNSs can effectively enhance the tensile strength and tensile elongation of titanium-based composites instead of 0.8 vol% BNNSs, as higher BNNS content results in agglomerated BNNSs and weakens the interface [21]. Similarly, Huang et al. [22] reported that excessive BNNSs lead to agglomeration, weakening the interface. Therefore, an appropriate amount of BNNSs need to be added into the Cu matrix to improve wear resistance.

In this paper, CMCs reinforced with BNNSs were fabricated by ball milling and spark plasma sintering (SPS). The microstructures of the composites were observed. The effects of the BNNS contents on the tribological behavior of the composites were investigated. Promising results were obtained, and the lubricating mechanism was discussed based on the characterizations of the worn surfaces.

## 2. Experimental Section

### 2.1. Raw Materials

Electrolytic copper powder (Cu, 99.9% purity) with a size of 20~40 μm was supplied by Jiangsu Men Da New Materials Technology Co., Ltd., Jiangsu, China, Hexagonal boron nitride (h-BN) powders with an average size of 10 μm were purchased from Beijing Xing Rong Yuan Technology Company, Beijing, China.

### 2.2. Preparation of Composites

**Figure 1:**
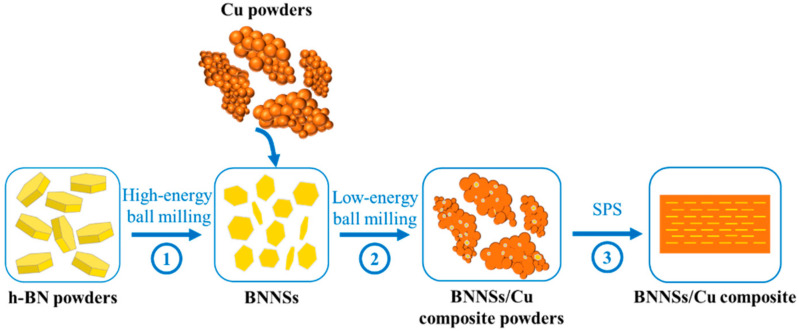
Schematic of fabrication process of BNNSs/Cu composite.

The fabrication process of CMCs is illustrated in Figure 1. BNNSs were prepared via high energy ball milling (HEBM, YXQM-2L, Changsha Miqi Instrument Equipment Co., Ltd., Changsha, China) with h-BN particles as raw materials. The as-received h-BN particles were milled with stainless steel balls with diameters of 5, 10 and 15 mm at 700 rpm for 40 min under an argon atmosphere, using alcohol as the dispersant. The ratio of the ball to powder was 40:1. The as-prepared solution was then dried at 60 °C for 24 h, allowing the alcohol to evaporate. The prepared BNNSs were mixed with Cu powder by low-energy ball milling at a rotational speed of 200 rpm for 30 min in stainless steel vials with stainless steel balls with diameters of 5 mm. Finally, the mixed BNNS/Cu composite powder was consolidated by SPS (Labox-325, SINTER LAND INC., Niigata, Japan) at 850 °C under a uniaxial pressure of 50 MPa for 10 min. The heating rate and cooling rate was 100 °C/min. Based on the above parameters, samples of pure Cu and composites with 0.2, 0.3, 0.5 and 0.8 vol% BNNSs were prepared and labeled as P-Cu, 0.2-CMC, 0.3-CMC, 0.5-CMC and 0.8-CMC, respectively.

### 2.3. Characterization

The morphologies of Cu powder, BNNSs/Cu mixture powder and the worn surfaces of BNNSs/Cu composites were examined using a field scanning electron microscope (FE-SEM, JSM7800F, JEOL Ltd., Tokyo, Japan) under an acceleration voltage of 15 KV and a working distance of 8 mm. The microstructure of the BNNSs and BNNSs/Cu composites were characterized by transmission electron microscopy (TEM, FEI Tecnai G2 F20, FEI Ltd., Hillsboro, OH，America) under an acceleration voltage of 100 KV. The surface roughness of the BNNSs/Cu composite samples was reduced by #5000 abrasive paper, and then they were polished with diamond slurry. Finally, the samples were etched with ferric chloride hydrochloric acid aqueous solution to observe their microstructures. The density was measure by the Archimedes method. Five samples for each BNNS content were measured to obtain the average density of the samples.

### 2.4. Microhardness Testing

The microhardness was tested with a Vickers hardness testing machine (HXS-1000TAC, Shoufeng Ltd., Shanghai, China) with a load of 100 g for 15 s according to the ASTM E384-11 standard [23]. Ten measurements were made from each sample for statistical analysis. In order to reduce the influence of surface roughness on microhardness, all the tested samples were polished carefully before the microhardness test.

### 2.5. Wear Testing

**Figure 2:**
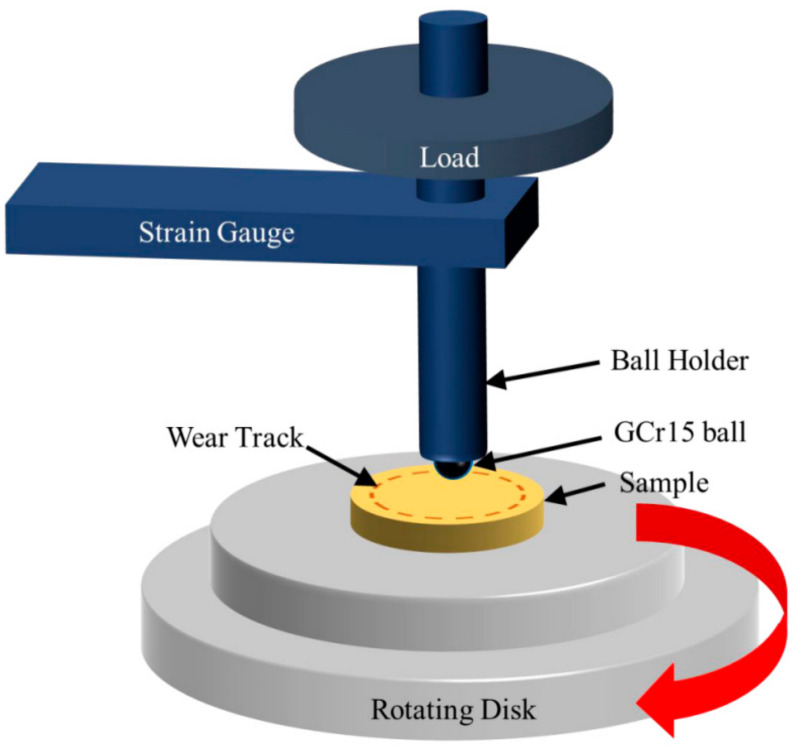
Schematic depicting the ball-on-disk friction test.

Dry wear tests were conducted on a ball-on-disk friction and wear tester (HT-1000, Zhongkekaihua Technology Development, Lanzhou, China, as schematically shown in Figure 2) at room temperature by sliding against a GCr15 ball with a diameter of 6 mm according to the ASTM G99 standard [24]. An amount of 3 N of normal force was applied over the ball by means of a set of weights. This way of applying loads ensures a stable force during the test. The sliding speed was 300 rpm with a friction radius of 3 mm. The sliding time was 10 min. The friction coefficient was directly measured using software supplied along with the machine. The wear rate *W* was calculated according to Equation (1) [25,26]:

(1) $$W=V∕\left(S\times L\right)\;$$

where S is the sliding distance (m), L is the load applied (N) and V is the wear volume (mm^3^). Three samples for each BNNS content were tested to obtain the average wear rate.

## 3. Results and Discussion

### 3.1. Morphology of Raw Materials

**Figure 3:**
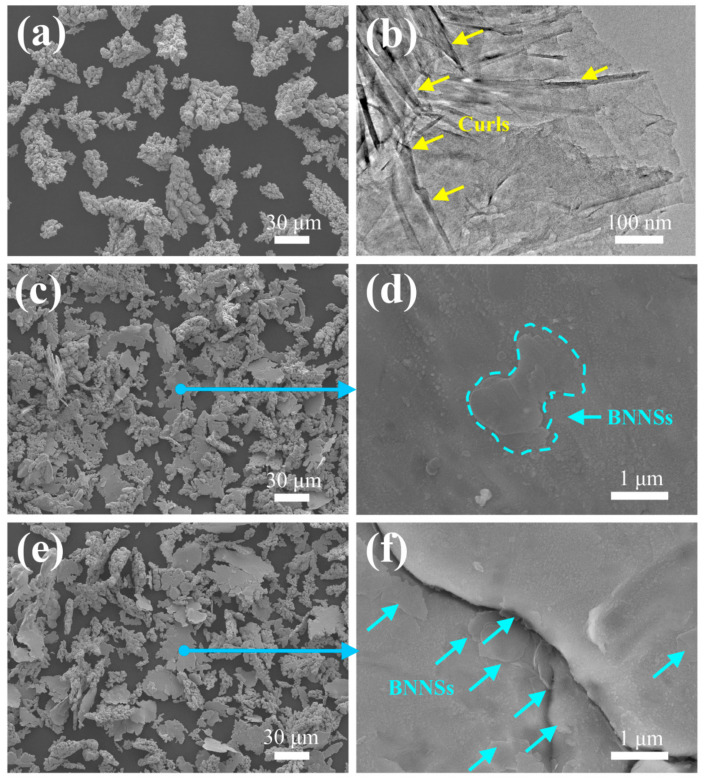
Powder morphology: (**a**) Cu powder (SEM), (**b**) BNNS powder (TEM), (**c**,**d**) 0.2 vol% BNNSs/Cu composite powder (SEM), (**e**,**f**) 0.8 vol% BNNSs/Cu composite powder (SEM).

Figure 3 shows the morphology of pure Cu particles, BNNSs and BNNSs/Cu composite particles. The pure Cu powder presents an irregular dendritic shape (Figure 3a). Figure 3b is the bright field transmission electron microscope (TEM) image of the BNNSs. They are so thin that they are electron-transparent. In addition, they are stacked with curls among them. The composite particles with 0.2 vol% BNNSs were flattened after the ball milling and BNNSs were homogeneously dispersed on their surface (Figure 3c,d). The composite particles with 0.8 vol% BNNSs resemble those with 0.2 vol% BNNSs. However, Figure 3f indicates that excessive BNNSs result in agglomeration, which can cause poor interfacial bonding and adversely affect the mechanical properties of the composites [20]. The fact that BNNSs are unfolded onto Cu particles evenly indicates that the ball milling process is suitable.

### 3.2. Characterization of the BNNSs/Cu Composites

**Figure 4:**
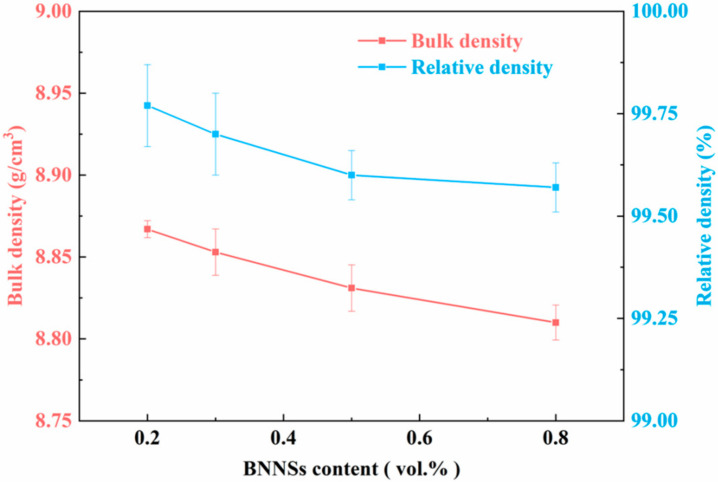
Bulk density and relative density: 0.2-CMC, 0.3-CMC, 0.5-CMC, 0.8-CMC.

Figure 4 shows the bulk density and relative density of BNNSs/Cu composites. It can be seen from Figure 4 that all the composite samples had a high relative density (over 99%). Such a high relative density can be attributed to the high ductility of Cu, synchronous uniaxial pressure and an electric current of SPS technology [27]. High temperatures soften the Cu particles, and the uniaxial pressure deforms them and makes them conform to each other. The current purifies the Cu surface, enabling inter-particle bonding. However, the relative density decreased with the reinforcement content. This is most likely because the rising BNNS content made the consolidation more difficult in the sintering process. The BNNSs hampered the deformation of the Cu matrix when the uniaxial pressure was employed.

**Figure 5:**
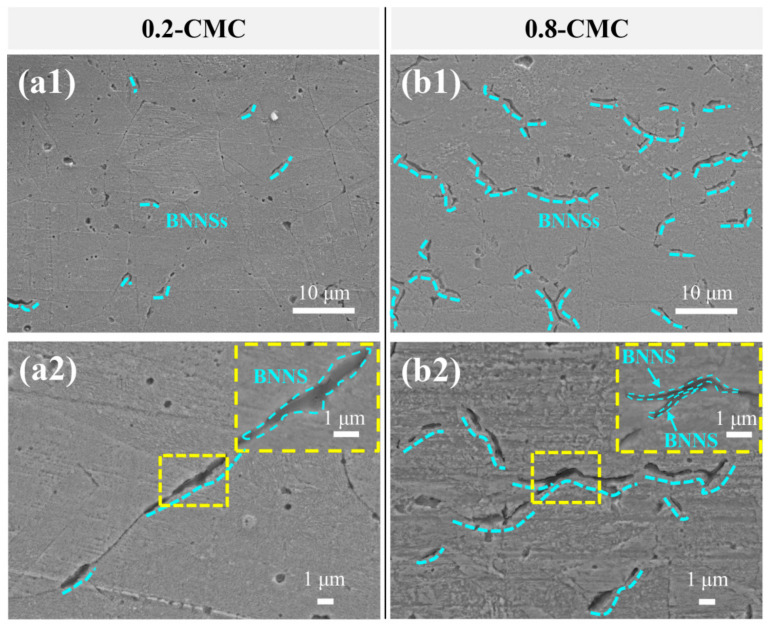
Microstructural characterization of the etched composite sample: (**a1**,**a2**) 0.2-CMC; (**b1**,**b2**) 0.8-CMC.

Figure 5 shows the cross-section SEM images of the etched surfaces of BNNSs/Cu composites. As shown in Figure 5(a1,b1), many BNNSs exist in long striped voids, which appeared after etching the cross-section. Therefore, the BNNS distribution characteristic of 0.2-CMC and 0.8-CMC can be distinguished. In Figure 5(a1), the content of the BNNSs is low, which ensures high bonding areas between Cu particles. The agglomeration of BNNSs was seldom observed (Figure 5(a1)). By contrast, the high BNNS content reduced the bonding area between Cu particles (Figure 5(b1)). In addition, Figure 5(b2) clearly shows the agglomeration of BNNSs. The properties of composites largely depend on their interface. If the interfacial strength is not strong, it becomes an origin of cracks. Specifically, if a high content of BNNSs cannot bond to adjacent Cu particles strongly, they cause them to peel off very easily. Moreover, the agglomeration of BNNSs also weakens the interface between the BNNSs and the Cu matrix. Therefore, a high BNNS content may be beneficial to the formation of self-lubricating film during the friction process. However, it inevitably induces cracks and peeling, causing serious surface damage.

**Figure 6:**
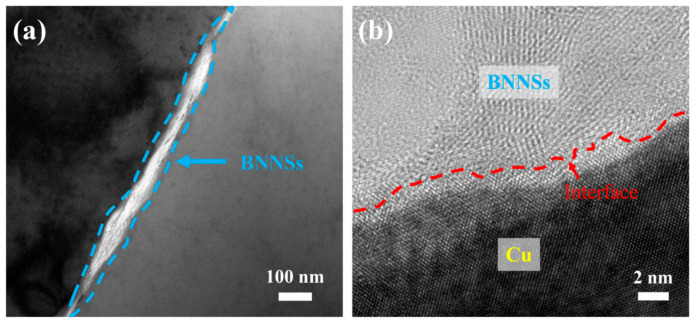
(**a**) TEM image and (**b**) HRTEM image showing the interface.

Figure 6 provides the TEM characterization of the interfacial region. No voids or gaps are observed in the BNNS–Cu interface (Figure 6a). This is in good agreement with the high relative density of the compacts. Figure 6b shows the HRTEM image of the interfacial microstructure. There is no impurity or transition layer in the interface. Therefore, Cu atoms formed metallurgical bonding with B and N atoms. In the sintering, due to its small atomic size, B could diffuse into Cu. Therefore, this interface can be viewed as one of diffusion, of which the bonding strength was medium [28,29].

### 3.3. Microhardness and Wear Resistance

**Figure 7:**
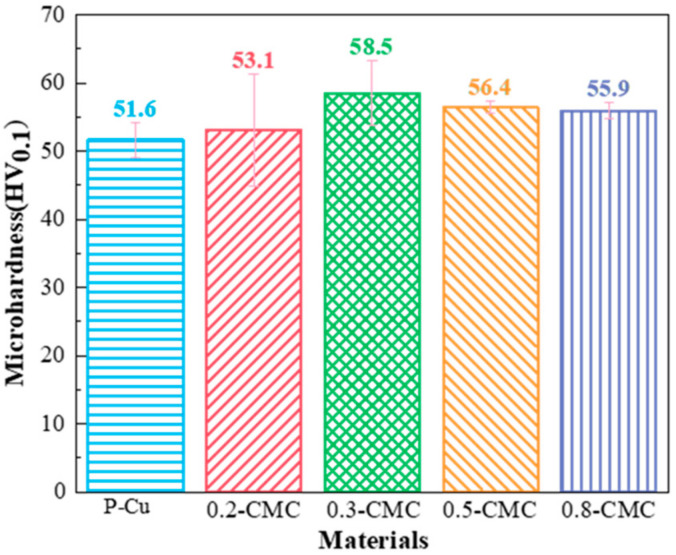
Microhardness of CMCs with different BNNS volume fractions.

Figure 7 shows the microhardness of P-Cu and the composites. As is apparent, the addition of BNNSs into Cu improved the microhardness; all the composites had a higher microhardness than P-Cu did. As the content of the BNNSs rose, the microhardness first increased then decreased, with 0.3-CMC possessing the highest microhardness of 58.5 HV_0.1_. The incorporation of BNNSs hindered the motion of dislocation, improving the microhardness, and excessive BNNSs led to conglomeration, weakening the interface and thus reducing the microhardness. As is apparent, the content and dispersion of BNNSs in Cu had a significant impact on the mechanical properties of BNNSs/Cu composites.

**Figure 8:**
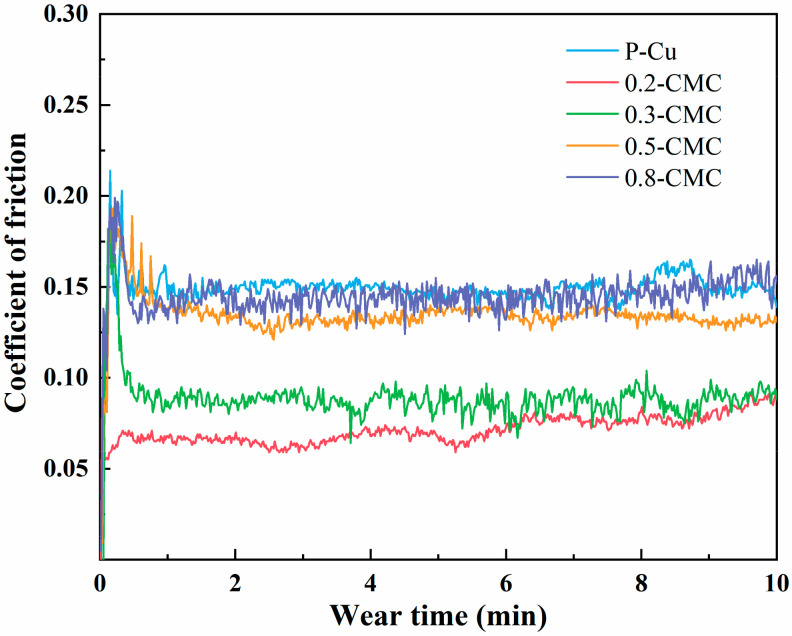
Coefficient of friction of CMCs with different BNNS volume fractions.

Figure 8 presents the friction coefficients of P-Cu, 0.2-CMC, 0.3-CMC, 0.5-CMC and 0.8-CMC. For all the samples, the friction coefficient decreased and stabilized after a rapidly increasing stage. However, the coefficients of friction (COF) of 0.2-CMC stabilized after a slowly increasing stage. Compared with CMCs, P-Cu had the highest average COF value of ~0.15, and 0.2-CMC, 0.3-CMC, 0.5-CMC and 0.8-CMC had average COF values of ~0.07, ~0.09, ~0.14 and ~0.15, respectively. It is worth mentioning that the addition of 0.2 vol% BNNSs considerably reduced the friction. However, “the more” does not mean “the better”. The average COF value of CMCs increased with the increase in the BNNS content, and the COF value of 0.8-CMC is close to that of P-Cu. According to previous studies [20,30], excessive nanosheets may reduce the properties of the composites.

**Figure 9:**
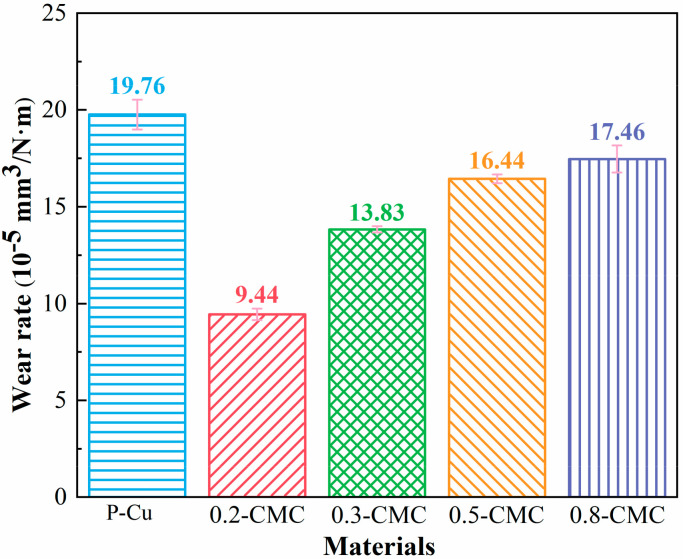
Wear rates of CMCs with different BNNSs volume fractions.

Figure 9 shows the corresponding wear rates of all samples. The 0.2-CMC possessed the lowest wear rate (9.44 ± 0.30) × 10^−5^ mm^3^/N∙m, which was merely 68%, 57%, 54% and 48% of 0.3-CMC, 0.5-CMC, 0.8-CMC and P-Cu, respectively. Specifically, within the scope of the present study, the addition of _BNNSs__ decreased the wear rate, and the addition of 0.2 vol% BNNS doubled the wear resistance. Like the role of the BNNS content in microhardness, “the more” does not mean “the better”. An amount of 0.2 vol% was the most suitable amount that was studied with respect to the wear resistance.

### 3.4. Wear Mechanisms of BNNSs/Cu Composites

**Figure 10:**
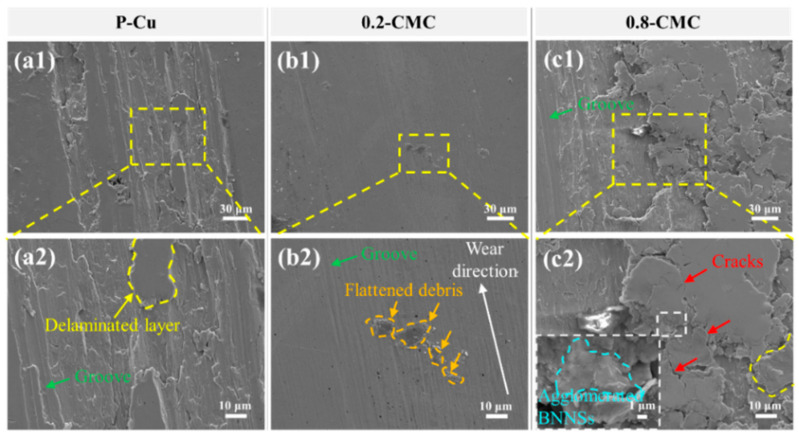
SEM images of the wear tracks: (**a1**,**a2**) P-Cu, (**b1**,**b2**) 0.2-CMC, (**c1**,**c2**) 0.8-CMC.

To explore the wear mechanisms of materials, the worn surfaces of P-Cu, 0.2-CMC and 0.8-CMC were characterized using FESEM (Figure 10). In Figure 10(a1,a2), only delaminated layers and grooves appear on the wear track of P-Cu. The delamination of surfaces indicates adhesive wear [31,32], which involves the initiation and propagation of cracks and a final fracture of the material in the near-surface region. Figure 10(b1) shows that 0.2-CMC had a much smoother surface than P-Cu did, and only sporadic debris and slight grooves were present on the worn surface. Some Cu debris was flattened in the wear process because of its low hardness. No cracks appeared in this wear track (Figure 10(b2)). This indicates that the dominant wear mechanism was abrasive wearing [33,34]. In contrast, 0.8-CMC exhibited a much coarser track than P-Cu and 0.2-CMC (Figure 10(c1)). Although slight grooves were also present, there were some delaminated flakes and agglomerated BNNSs (Figure 10(c2)). In the composites with high BNNS content, the medium interfacial bonding strength between Cu and BNNSs and the agglomeration of BNNSs enabled Cu particles to be easily peeled by the sliding ball.

BNNSs are the key to the major wear mechanisms of the composites. They can have complicated effects on the wear. First, the introduction of BNNSs improves the hardness, making the penetration of the sliding ball more difficult and thus reducing the depth of the ploughing grooves [19]. Second, BNNSs can act as a solid lubricant, hence preventing the direct metal-to-metal contact and improving the wear resistance [35]. Third, BNNSs may become the origin source of cracks due to their medium bonding strength with the Cu matrix. Finally, agglomerated BNNSs can weaken the interfacial bonding between adjacent composite particles, making some composite particles easily peeled off [36,37]. In short, BNNSs may have both positive and negative effects on the wear resistance of CMCs.

The overall effects of BNNSs in wear resistance are determined by their contents and dispersion. In 0.2-CMC, BNNSs were uniformly dispersed in the matrix without agglomeration. They improved the microhardness and serve as the lubricator. Therefore, the positive effects on the wear resistance were dominant. In 0.8-CMC, by contrast, the agglomeration of the BNNSs was evident. BNNSs also increased the microhardness, but to a lower extent. They still served as a lubricator. However, their agglomeration makes the adjacent Cu particles easily peeled off by the sliding ball [38]. This means that, in 0.8-CMC, the aiding effects of BNNSs were weakened by the adverse effects of their agglomeration. This is the reason why the wear resistance of 0.8-CMC was superior to P-Cu but inferior to 0.2-CMC.

## 4. Conclusions

In this work, BNNSs/Cu composites were consolidated using SPS. The relative density of the composites was over 99%, and it decreased with BNNS content. The introduction of 0.2 vol% BNNSs can effectively reduce the coefficient of friction and the wear rate. The coefficient of friction of 0.2-CMC was merely 0.07, compared with 0.15 of P-Cu, and the wear rate of 0.2-CMC was reduced by about 52% compared with P-Cu. Excessive amounts of BNNSs had an adverse effect on the wear resistance. The 0.8-CMC had a higher wear rate than 0.2-CMC did. The addition of BNNSs altered the wear mechanisms. Evident delamination was found on the surface of P-Cu after the wearing process, and no evident cracks or delamination were present in 0.2-CMC. In 0.8-CMC, the wear track was much coarser, as excessive BNNSs can weaken the interfacial bonding, making composite particles peeled off more easily.

## Data Availability

The datasets generated and/or analyzed during the current study are available from the corresponding author upon reasonable request.
